# The mediating role of self-control on the relations between adverse childhood experiences and substance use among adolescents in Uganda

**DOI:** 10.3389/fpsyg.2024.1297565

**Published:** 2024-05-14

**Authors:** Jane Namusoke, Kennedy Amone-P’Olak, Carol Chosen Nakanwagi, Henry Kibedi, Nathaniel Mayengo, Joseph Ssenyonga, Bernard Omech

**Affiliations:** ^1^Department of Psychology, Kyambogo University, Kampala, Uganda; ^2^Department of Foundations of Education and Educational Psychology, Kyambogo University, Kampala, Uganda; ^3^Directorate of Graduate Training and Research, Lira University, Lira, Uganda

**Keywords:** adverse childhood experiences, substance use, self-control, adolescents, Uganda

## Abstract

**Objective:**

Adverse childhood experiences (ACEs) are established risk factors for undesirable consequences in adolescence and early adulthood, including substance use and a lack of self-control. Based on the Social Bonds Theory (SBT), this study aims to expand our knowledge of the pathways from ACEs and self-control to substance use in adolescence and early adulthood.

**Methods:**

The extent to which self-control mediates the association between ACEs and substance use was examined in a cross-sectional survey of 358 adolescents and young adults (*N* = 234, 65.5% girls, mean age 17.7, *SD* 0.58, *range* 15–18). Data were gathered using the Adverse Childhood Experiences (ACE-10) questionnaire, the Drug Abuse Screening Test (DAST-10), and the 10-item self-control scale to assess childhood adversity, substance use, and self-control, respectively.

**Results:**

ACEs were widely reported and significantly associated with substance use and a lack of self-control. Self-control strongly predicted substance use, independent of ACEs. Among those reporting no ACEs, one to two, three to four, and five or more, there were significant variations in the respondents’ substance use (*F*_(3, 400)_ = 12.69, *p* = 0.001). Self-control explained 51.2% (95% confidence interval [CI]: 41, 61%) of the associations between ACEs and substance use as assessed by linear regression.

**Conclusion:**

Self-control is key to understanding why adolescents and young adults with a history of childhood adversity indulge in substance use. Therefore, there is a need to advocate for psychological interventions such as cognitive and behavioural therapy that have demonstrated efficacy in promoting self-control in adolescents and young adults.

## Introduction

In sub-Saharan Africa, including Uganda, substance use is a major public health concern with 41.6 percent of adolescents and young adults using substances (Olawole-Isaac et al., 2018). Substance use is defined as the use of specific substances, such as alcohol, tobacco products, inhalants, and other chemicals that could be absorbed by the body through injection, inhalation, or other means that may cause dependence or other negative effects [National Center for Health Statistics (US), 2023, p. 26]. A study in Uganda found that substance use is common and widespread among adolescents and young adults, particularly those in secondary and tertiary education institutions (Kaggwa et al., 2022). Moreover, the study found that the most popular substances used included the alcoholic beverage vodka (23.3%), Kuber (10.8%), Khat (10.5%), aviation fuel (10. 1%), cannabis (9.2%), and cigarettes (5.9%), in that order (Abbo et al., 2016; Kaggwa et al., 2022).

Early age of onset (e.g., early adolescence) of any drug and substance is known to be associated with elevated risk and faster transition to substance use disorders (Behrendt et al., 2009). Moreover, adolescents and young adults who use substances are at risk for long-term problems such as dropping out of school, criminal behaviours, and a life of poverty (Olawole-Isaac et al., 2018). Additionally, substance use exposes adolescents and young adults to risky behaviours as well as a variety of illnesses [e.g., HIV/AIDS, Sexually Transmitted Infections (STIs), motor accidents, and self-harm; Breet et al., 2018a,b; Kaggwa et al., 2022; Kwagala et al., 2022; Mavura et al., 2022]. A variety of developmental abnormalities in neurotransmission have also been linked to substance use in adolescents and young adults, as well as long-lasting neurobiological alterations in the Hypothalamus-Pituitary-Adrenocortical (HPA) axis development and function that is related to stress control and regulation (Thorpe et al., 2020).

Further, it is well documented that there are gender and sex differences in substance use, with men using drugs and substances at higher rates than women (McHugh et al., 2019). Gender and sex differences are mostly the result of two factors: socio-cultural and biological. While socialisation and access to drugs and substances are major drivers of gender variations in drug and substance use in the socio-cultural domain, biological factors such as brain structure and function, endocrine systems, and metabolic processes are important determinants of biological differences in men and women (McHugh et al., 2019). In Sub-Saharan Africa, substance use behaviour is more prominent in males than females (Fentaw et al., 2022). The lifetime and current substance use were 3.2 and 2.8 times higher among males compared to females (Fentaw et al., 2022). Furthermore, sex and gender variations in substance use are known to exist with more use in men than women (McHugh et al., 2019; Nishimura et al., 2022; Oguntayo et al., 2022). Two major factors underlie sex and gender differences: biological and sociocultural. Biologically, brain structure and function, endocrine functions, and metabolic functions are key determinants of biological differences (McHugh et al., 2019) while socialisation and access to drugs and substances are key drivers of gender variations in drug and substance use (Oguntayo et al., 2022).

Therefore, understanding drug and substance use among the adolescent subpopulation is essential for guiding practice and policy. Additionally, the evidence base for targeted preventative interventions aimed at reducing substance use and abuse may be provided by knowledge of substance use in this subpopulation.

Substance abuse has been associated with several factors, such as temperament, neighbourhoods, and ACEs (Ludick and Amone-P’Olak, 2016; Oguntayo et al., 2022). One factor that needs to be explored further is exposure to adverse childhood experiences (ACEs) about substance abuse. A variety of other familial characteristics have been recognised as drivers of substance use in adolescents and young adults. These characteristics include a history of mental health problems in the family, familial substance use, separations and divorce, emotional and physical abuse and neglect, sexual abuse, violence towards mother or stepmother, incarceration of a family member, and other psychosocial challenges linked to family instability and dysfunction (Abbo et al., 2016; Mongale and Amone-P’Olak, 2019; Engebretsen et al., 2020; Ramotuana and Amone-P’Olak, 2020; Rukundo et al., 2020; Kaggwa et al., 2022). These negative familial characteristics are generally categorised as “Adverse Childhood Experiences” (ACEs).

ACEs have also been linked to impaired self-control or ability to self-regulate and the inability to anticipate the long-term effects of one’s actions (Gottfredson and Hirschi, 1990). The inability to self-regulate is manifested by characteristics such as “impulsiveness,” “self-centeredness,” “risk-seeking,” and “pursuit of instant rewards and benefits” (Gottfredson and Hirschi, 1990). This lack of competence to self-regulate is a result of the characteristics the person may carry as a result of negative familial characteristics occasioned by ACEs. Often, a person who lacks self-control prefers instant pleasure, easy activities with little to no planning, without anticipation of negative consequences of their actions, little to no empathy for other people, and is prone to deviant behaviours (Gottfredson and Hirschi, 1990). As a result of these characteristics, many adolescents and young adults are more likely to abuse drugs and substances. In addition, ACEs can alter brain morphology and function, particularly in the medial prefrontal and hippocampal regions, which can impact the ability for self-regulation (Lackner et al., 2018). For adolescents and young adults to resist urges to engage in deviant behaviours like substance use, self-regulatory abilities are essential.

Prior research has mostly focused on the connections between ACEs and substance use in the subpopulation of adolescents and young adults without taking into account the possibility that various ACEs and cumulative trauma may have diverse effects on substance use (Shin et al., 2018; Rogers et al., 2022). It is unclear, for instance, which ACE categories have a more harmful influence or whether it is the confluence of the ACEs that are associated with drug and substance use. Even though ACEs are known to impact substance use, similar connections are known to exist between ACEs and self-control (Meldrum et al., 2020). The ability to put off immediate gratification in favour of long-term gains is a common indicator of self-control and the primary motivator of deviant activity (Gottfredson and Hirschi, 1990).

Adolescents and young adults are more likely to engage in behaviours that result in immediate rewards and benefits, such as substance abuse, when they exhibit signs of poor self-control, such as impulsivity, self-centeredness, and risk-taking (Gottfredson and Hirschi, 1990). As a result, adolescents and young adults with greater degrees of self-control are less likely to abuse substances (Meldrum et al., 2020). Consequently, we hypothesise that exposure to various childhood adversities may be linked to substance use in adolescence and early adulthood.

The specific objectives were fourfold: (1) to evaluate the students’ reports of ACEs, substance use, and self-control, (2) to assess the differential influence of individual, categorical and cumulative ACEs on substance use in univariable regression models, (3) to assess the influence of self-control on substance use in a univariable regression model, and (4) to evaluate the mediating effect of self-control in the relationship between ACEs and substance use in multivariable regression models.

### Theoretical underpinning

According to the Social Bonds Theory (SBT), if people are not controlled, they will engage in abnormal behaviours (Hirschi, 1969a,b). This idea contends that people who have close ties to their families, schools, or other institutions are less likely to participate in inappropriate behaviours (Hirschi, 2002). These social bonds are anchored on four social pillars, namely: *attachment*, *commitment*, *involvement*, and *belief*. Attachment is the psychological respect and regard that a person has for institutions and others (Hirschi, 1969a,b). Additionally, *attachment* strengthens moral character and prevents people from engaging in antisocial behaviour (Stewart, 2003). Likewise, the ability to pursue objectives, such as those related to school or employment, shows *commitment*, the second anchor of the SBT (Hirschi, 1969a,b). As a result, commitment to these goals will prevent a person from engaging in actions that will impede the achievement of such goals. Besides, devoting one’s time and energy is what is referred to as *involvement*, which is the third anchor of the Social Bonds Theory (Hirschi, 1969a,b). An individual is less likely to participate in deviant behaviours or be negatively influenced by others if they invest more time and effort into an activity, such as schoolwork (Hoeben and Weerman, 2016). *Belief* is the final tenet of SBT proposed by Hirschi, which is the conviction that rules and regulations are morally valid. People who strongly believe that an institution’s rules and regulations are ethically valid and need to be obeyed are less likely to engage in behaviour that differs from that of the institution. Positive perceptions of institutional norms, rules, and regulations consequently influence their compliance with the institution’s rules and regulations and law-abiding and prosocial behaviour.

In summary, SBT can offer a conceptual framework for explaining the links between ACEs, substance use, and the possible mediating effect of self-control in the relations between ACEs and substance use. This is made possible by the focus on the value of social relationships (attachment, commitment, engagement, and belief) and how ACEs can weaken these bonds, raising the likelihood of indulging in substance use. Poor self-control erodes the capacity of adolescents and young adults to resist instant gratification and uphold stronger social bonds. Consequently, we hypothesised that self-control mediates the associations between ACEs and substance use in adolescence and early adulthood.

## Methods

### Design and sample

This study utilised a cross-sectional design. The sample size was computed based on regression statistical analyses using G*Power 3.1.9.2 software (Faul et al., 2009). With an effect size of 0.8, a significance level of α = 0.05, and a statistical power of 1-β = 0.8, the power analysis showed a sample size of 350 respondents. The sample size was determined *a priori*. A random cluster sampling technique was employed to select respondents from a group of adolescents enrolled in eight secondary schools located in Kampala, the capital city of Uganda. Each cluster comprised boarding and day schools. The schools were selected in a ratio of one-to-one (1:1) as recommended by Cone and Foster (2008). Students in the third through fifth years of study (9th through 12th grade of formal school) were chosen at random from eight secondary schools in three clusters: boarding schools (one boarding and 1 day); secondary schools for boys only (one boarding and 1 day); mixed schools (one boarding and another day); and secondary school for girls only (one boarding and another day). Within the schools, we used a sampling frame to draw 60 male and female respondents from each class in a ratio of one to one (1:1). Subsequently, a total of 358 respondents were invited to participate in the study.

### Instruments

The questionnaire for this study was made up of three sections: a list of sociodemographic characteristics, ACEs, social control, and substance abuse.

*Socio-demographic characteristics*: Age, academic year, gender, and place of upbringing (such as a rural or urban environment) were among the socio-demographic factors measured.

*Adverse Childhood Experiences (ACEs) questionnaire*: The ACEs questionnaire assesses past experiences of emotional abuse, physical abuse, emotional neglect, physical neglect, sexual assault, household substance abuse, household mental illness, parental separation or divorce, violent treatment of the mother, and incarceration of a household member (Felitti et al., 1998; Finkelhor et al., 2015). Examples of questions on the ACEs questionnaire are “Were your parents ever divorced or separated?” and “Were you ever hit, beat, kicked, or physically hurt in any way by a grown-up in your life, excluding spanking on your bottom?” The responses were dichotomously scored as “yes” (= 1) for occurrence and “no” (= 0) for non-occurrence. The scores on individual items of the ACEs questionnaire were summed to indicate an aggregate score for the variable ACEs. A higher score on the ACEs questionnaire indicated severe adversity (score *range* = 0–10). The reliability of the 10-item questionnaire and internal consistency calculated using the Kuder–Richardson Formula 20 (KR-20) were acceptable in this study at 𝛂 = 0.75. The 10-item ACE questionnaire has been utilised in the past in this population and others with acceptable levels of internal consistency (Naicker et al., 2017; Manyema and Richter, 2019; Amone-P’Olak and Letswai, 2020).

*Substance use*: The Drug Abuse Screening Test (DAST-10) developed by Skinner in 1982 was used to evaluate the use of a variety of substances, including cocaine, cannabis (marijuana, hashish), solvents (petrol, paint thinner), tranquillizers (Valium), barbiturates, speed, methamphetamines, and hallucinogens (LSD), as well as narcotics (heroin) and tranquillizers (Valium). Previous studies (Yudko et al., 2007; Benschop et al., 2015) have established the psychometric properties of the DAST-10 questionnaire. The internal consistency in this study, as determined by the Kuder–Richardson Formula 20 (KR-20), was 𝛂 = 0.73. Questions about alcohol and cigarette usage were added to the DAST-10 survey.

*Social Control Questionnaire:* The 10-item social control questionnaire developed by Tangney et al. (2004) was used to measure self-control among adolescents and young adults. Items were scored on a Likert-type scale with response options ranging from 1 to 5 with 1 = “very much like me” to 5 = “not at all like me” for the items 1, 2, 3, 7, 8, 9, and 10, while the remaining items were reverse-scored. Individual item scores were summed up and divided by 10. The maximum score on the scale is 5 (extremely self-controlled) and the minimum score is 1 (not at all self-controlled). The internal consistency reliability was acceptable at 𝛂 = 0.78 (based on the data so far entered).

To ascertain the validity of the instruments, Principal Component Analyses (PCA) were utilised as the extraction method. The best fit for the data was a one-factor solution for each of the instruments that explained 69.1, 65.7, and 63.5% of the variance for the ACE-10, DAST-10, and Social Control Questionnaire, respectively. The best-fit parameters were obtained from a PCA based on a direct obliging rotation technique with a cut-off of 0.30 using Kaiser’s (1960) criterion of eigenvalues.

### Procedure

To obtain authorization to gather data from students while they were in class, school officials and teachers from different schools were approached. Classes were chosen at random from each school’s teaching timetable before reaching out to administrators and teachers. Before gathering data, the student’s consent or assent was sought. The study’s goal, the student’s right to decline or withdraw at any time, and the confidential nature of their participation were all explained to them before they gave their consent. The students were asked not to provide any identifying information on the questionnaire to remain anonymous. In the presence of a research assistant in the class to address any questions the students may have; it took the students between 10 and 15 min to complete the questionnaire. A total of 400 students who were enrolled in various schools at various stages of the study took part in the study. Some questionnaires were disregarded from the analyses due to their respondents’ advanced ages (students older than 18 years, for example). Finally, analysis was performed on data from 358 students (65.5% female, *n* = 234) with a mean age of 16.3 years (SD = 0.88, Range = 15–18).

### Ethical considerations

Lira University Research Ethics Committee gave its ethical approval for the study (LU/2023/0029). To obtain authorization to gather data from students while they were in class, school officials and teachers from different schools were approached. Classes were chosen at random from each school’s teaching timetable before reaching out to administrators and teachers. Before gathering data, the student’s consent or assent was sought. The study’s goal, the student’s right to decline or withdraw at any time, and the confidential nature of their participation were all explained to them before they gave their consent. The students were asked not to provide any identifying information on the questionnaire to remain anonymous. In case of any psychological problems experienced during the research, the students were immediately notified of the availability of free psychological services at *Safe Places* and *Mental Health Uganda*, both of which are based in Kampala.

### Data analysis

Data from secondary school students taking part in the ongoing project “Childhood Adversity and Substance Use in Adolescence and Early Adulthood in Uganda” were used to examine the impact of both total and categorical ACEs on substance use. From this data, the students’ reports of ACEs, substance use, and self-control were assessed using descriptive statistics (mean, SD, and range). Next, univariable regression models were fitted to ascertain the extent to which individual and cumulative ACEs and self-control predicted substance use. Further, while controlling for sociodemographic factors including age and sex, a multivariable regression model was fitted to examine whether ACEs predicted substance use individually and cumulatively to ascertain their unique and independent influences. In addition, the extent to which self-control (a continuous variable) predicted substance use was assessed in a multivariable regression analysis while adjusting for sociodemographic characteristics like age and sex. Finally, different categories of ACEs scores (i.e., “0,” “1–2,” “3–4,” and “5″ and above) were used as the independent variable in a one-way analysis of variance (ANOVA), and substance use (i.e., “0,” “1,” “2–3,” and “4″ and above) as the dependent variable. Additionally, the cumulative score on ACEs in the multivariable analysis was computed, and the effect sizes of each ACE in univariable and multivariable models were calculated using Eta squared (η^2^), where η^2^ ≥ 0.01, η^2^ ≥ 0.06, and η^2^ ≥ 0.14 were considered to represent small, moderate, and large effect sizes in that order. To check for differences between levels of ACEs severity, the Tukey HSD test for multiple comparisons was utilised.

The framework developed by Baron and Kenny (1986) was used to determine the extent to which self-control (a continuous measure) mediated the associations between ACEs (a continuous measure) and substance use (an aggregate score). Mediation analysis is appropriate for this study since the ACEs assessed occurred before the age of 18, and self-control and substance use were evaluated for their current occurrence. As a result, this study’s temporal order of events—a requirement for mediation analyses—meets the criteria for mediation analyses. Both predictor and mediator variables were standardised to a mean of zero (“0”) and a standard deviation of one (“1”) [Z scores]. In addition, bootstrapping methods were run to obtain 95% confidence limits (95% Confidence Interval [CI]) for the mediated effects. Confidence intervals based on bias-corrected bootstrapping are generally preferred (Cheung, 2007). Next, mediation was assessed by determining the degree of attenuation in the relation between ACEs and substance use after including self-control as a covariate. The attenuation was scaled as the relative decrease in the regression coefficient for ACEs. IBM SPSS statistical software, version 29.0 (IBM Corp, 2021), was used for all statistical analyses. Associations were deemed statistically significant if their *p*-value was less than 0.05.

## Results

### Sociodemographic characteristics

Tables 1, 2 summarise the overall sociodemographic characteristics of the participants in the study. Data were gathered from 358 students (*n*,%) with an average age of 16.3 (±0.88, range: 15–18). Male (Mean age = 17.26, 0.76) students were significantly older than their female (Mean age = 16.83 ± 0.90) counterparts (*t* = 4.57, *p* < 0.01).

**Table 1:** Prevalence of individual ACEs and the total number of ACEs reported by participants (*N* = 358).

	Total		Male		Female		T-test	Cohens D
Variables	*M (± SD)*	*N* or *n (%)*	*M (±SD)*	*N* or *n (%)*	*M (±SD)*	*N* or *n (%)*		
**Prevalence of individual ACEs**								
1. Emotional abuse		150/357 (42)		50/123 (40.7)		100/233 (42.9)	ns	
2. Emotional neglect		129/353 (36.5)		35/122 (28.7)		94/230 (40.9)	**−2.32, *p* < 0.05**	**0.38**
3. Physical abuse		115/356 (32.3)		49/122 (40.2)		66/233 (28.3)	**2.27, *p* < 0.05**	**0.33**
4. Separation/Divorce		89/352 (25.3)		31/121 (25.6)		58/230 (25.2)	ns	
5. Household substance abuse		77/357 (21.6)		28/123 (22.8)		49/233 (21.0)	ns	
6. Incarcerated household member		70/349 (20.1)		25/120 (20.8)		45/228 (19.7)	ns	
7. Mother treated violently		50/357 (14.0)		21/123 (17.1)		29/233 (12.4)	ns	
8. Household mental illness		50/357 (14.0)		13/123 (10.6)		37/233 (15.9)	ns	
9. Sexual assault		48/354 (13.6)		8/122 (6.6)		40/234 (17.3)	**−3.20, *p* < 0.01**	**0.42**
10. Physical neglect		24/354 (6.8)		12/121 (9.9)		12/232 (5.2)	ns	
**Number of ACEs reported**								
0		66/358 (18.4)		27/123 (22.0)		38/234 (16.2)		
1		78/358 (21.8)		24/123 (19.5)		54/234 (23.1)		
2		75/358 (20.9)		24/123 (19.5)		51/234 (21.8)		
3		59/358 (16.5)		19/123 (15.4)		40/234 (17.1)		
4		36/358 (10.1)		14/123 (11.4)		22/234 (9.4)		
≤ 5		44/358 (12.3)		15/123 (12.2)		22/234 (12.4)		
Total number of ACEs reported	2.24 (±1.83)		2.21 (±1.88)		2.27 (±1.81)		ns	

ACEs, adverse childhood experiences; M, mean; N, total number of participants; n, sub-population; SD, Standard deviation; %, percent. Significant results are indicated in bold.

**Table 2:** Prevalence of individual Substance stratified by gender (*N* = 358).

	Total		Male		Female		T-test	Cohens D
Variables	*M* (*±SD*)	*N* or *n (%)*	*M* (*±SD*)	*N* or *n (%)*	*M* (*±SD*)	*N* or *n (%)*		
**Prevalence of individual substance use**								
Alcoholic drink		187/358 (52.5)		66/123 (53.7)		121/232 (52.2)	ns	
Codeine or Cough syrups		114/358 (31.8)		25/123 (20.3)		88/234 (37.6)	**−3.57, *p* < 0.01**	**0.44**
Cigarette or tobacco		49/357 (13.7)		25/122 (20.5)		24/234 (10.3)	**2.68, *p* < 0.05**	**0.36**
Marijuana, weed, or cannabis or banghi?		33/356 (9.3)		16/123 (13.0)		17/232 (7.3)	**2.00, *p* < 0.05**	**0.30**
Inhalants, e.g., jet fuel, petrol, diesel, thinner, glue, etc.		25/355 (7.0)		7/123 (5.7)		18/231 (7.8)	ns	
Cozepam, Benzho, Diazepam, Valium, etc.,		21/356 (5.9)		4/123 (3.3)		17/232 (7.3)	**−2.05, *p* < 0.05**	**0.33**
Mairungi, Khat, or Miraa		20/356 (5.6)		11/122 (9.0)		9/233 (3.9)	**2.02, *p* < 0.05**	**0.32**
Opium or heroin		7/356 (2.0)		5/122 (4.1)		2/233 (0.9)	**2.03, *p* < 0.05**	**0.32**
Cocaine, White powder, or Crack		15/357 (4.2)		6//123 (4.9)		9/233 (3.9)	ns	
Other drug(s)		25/331 (7.6)		11/118 (9.3)		14/212 (6.6)	ns	
**Number of drug use reported**								
0		121/358 (33.8)		40/123 (32.5)		81/234 (34.6)		
1		106/358 (29.6)		38/123 (30.9)		67/234 (28.6)		
2		62/358 (17.3)		18/123 (14.6)		44/234 (18.8)		
3		38/358 (10.6)		14/123 (11.4)		24/234 (10.3)		
≤ 4		31/358 (8.7)		13/123 (10.5)		18/234 (7.7)		
Total number of drug use reported (*M*, ± *SD*)	1.66 (±1.51)		1.78 ± 1.51		1.52 (±1.51)		**1.99, *p* < 0.05**	**0.30**

ACEs, adverse childhood experiences; M, mean; N, total number of participants; n, sub-population; SD, Standard deviation; %, percent. Significant results are indicated in bold.

The majority of the students (*n* = 292, 81.6%) reported at least one or more ACEs. The total number of ACEs reported showed no gender differences. However, the reporting of particular ACEs, such as sexual abuse, emotional neglect, and physical abuse, differed by gender. Male students reported more physical abuse than female students, but female students reported significantly more emotional neglect and sexual abuse (Table 1). The most frequently reported ACEs were: emotional abuse, emotional neglect, physical abuse, separation and divorce, and household substance abuse, in that order.

The majority of the students (*n* = 237, 66.2%) surveyed admitted using one or more substances. The most often reported substance use (*n* = 187, 52.5%) was alcohol followed by cigarettes, cough syrup containing codeine, and marijuana, in that order. There were no differences in the use of alcohol, cocaine (white powder or crack), and inhalants (such as jet fuel, petrol, diesel, glue, or thinner) between male and female students. Compared to male students, female students used benzodiazepines, valium, codeine, and cough syrup significantly more than male students. On the contrary, male students significantly used more cigarettes, mairungi (also known as khat), marijuana, and opium than female students (Table 2).

Overall, male students reported using substances significantly more frequently than their female counterparts (*t* = 4.57, *p* < 0.01). However, regarding ACEs and self-control, there were no significant differences between male and female students. In summary, increased substance use and higher ACEs, low self-control, and being a boy were generally associated with substance use.

**Figure 1:**
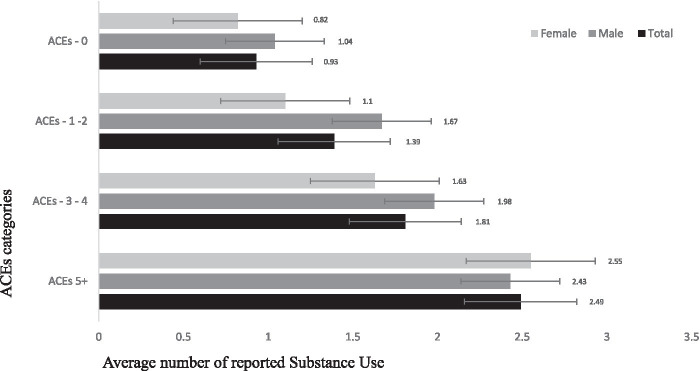
Reports of substance use stratified by ACEs categories.

The bar graph shows substance use stratified by various categories of ACEs reported (categories include “0” for no ACEs, “1–2,” “3–4” ACEs, and “5” ACEs). Overall, as more ACEs were reported, the number of substance use reported gradually increased (Figure 1).

### The influence of ACEs on substance use in univariable and multivariable regression models

Generally, the total number of ACEs significantly predicted substance use as a continuous measure (*β* = 0.41, 95% confidence interval [CI]: [0.30, 0.52]). Household mental illness, sexual abuse, household substance abuse, incarceration of household members, and physical abuse and neglect, in separate univariable regression models, significantly predicted substance use (Table 2). Similarly, the three categories of ACEs: household dysfunction, abuse, and neglect, in separate univariable regression models, also significantly predicted substance use (Table 3). The regression model yielded a significant fit (*R*^2^ = 0.16, *F*_(2, 356)_ = 56.43, *p* < 0.001). However, the regression model did not improve with the inclusion of sex as a variable. Each regression coefficient is the ratio of the SD change in the predictor variable to the SD change in the outcome variable. For instance, the regression of substance use on the total number of ACEs is indicative of a change of 1 SD related to a change of 0.41 SD in substance use.

**Table 3:** Univariable regression analyses of the influence of individual and total number of ACEs based on a continuous measure of the total number of reported substance use in the past year and current Self-control.

	Drug and substance use		Self-control	
Variables	*β* [95% CI]	η^2^	*β* [95% CI]	η^2^
**Individual ACEs**				
1. Household mental illness	**0.20 (95% CI: 0.09, 0.30)**	**0.05**	**−0.12 (95% CI: −0.22, −0.02)**	**0.01**
2. Sexual assault	**0.14 (95% CI: 0.04, 0.25)**	**0.02**	**−0.15 (95% CI: −0.25, −0.05)**	**0.03**
3. Incarcerated household member	**0.14 (95% CI: 0.04, 0.25)**	**0.02**	0.03 (95% CI: −0.08, 0.13)	0.00
4. Physical neglect	**0.13 (95% CI: 0.02, 0.23)**	**0.02**	−0.03 (95% CI: −0.13, 0.08)	0.00
5. Physical abuse	**0.13 (95% CI: 0.02, 0.23)**	**0.02**	**−0.16 (95% CI: −0.27, −0.06)**	**0.03**
6. Emotional neglect	0.10 (95% CI: −0.01, 0.21)	0.01	**−0.18 (95% CI: −0.28, −0.07)**	**0.04**
7. Household substance abuse	0.10 (95% CI: −0.01, 0.21)	0.01	−0.09 (95% CI: −0.20, 0.01)	0.00
8. Emotional abuse	0.10 (95% CI: −0.01, 0.20)	0.01	**−0.17 (95% CI: −0.27, −0.07)**	**0.04**
9. Mother treated violently	0.05 (95% CI: −0.06, 0.15)	0.00	**−0.14 (95% CI: −0.24, −0.04)**	**0.02**
10. Separation/Divorce	0.04 (95% CI: −0.09, 0.22)	0.00	−0.03 (95% CI: −0.13, 0.08)	0.00
**Three categories of ACEs**				
1. Household dysfunction	**0.19 (95% CI: 0.09, 0.29)**	**0.04**	**−0.12 (95% CI: −0.23, −0.02)**	**0.01**
2. Abuse	**0.19 (95% CI: 0.08, 0.29)**	**0.04**	**−0.24 (95% CI: −0.34, −0.14)**	**0.06**
2. Neglect	**0.14 (95% CI: 0.03, 0.24)**	**0.03**	**−0.16 (95% CI: −0.26, −0.05)**	**0.03**
Total number of ACEs	**0.24 (95% CI: 0.14, 0.34)**	**0.06**	**−0.23 (95% CI: −0.33, −0.13)**	**0.05**

*β*, Standardised beta; CI, Confidence Intervals; η^2^, Eta Squared (a measure of effect size). All significant associations are in bold.

Only household mental illness, household substance use, and sexual abuse remained significant when all the ACEs were concurrently added to a single regression model to evaluate their unique influences on substance abuse (Table 4). Similarly, for the three categories of ACEs (neglect, abuse, and abuse), only abuse and household dysfunction remained significant predictors of substance use (Table 3).

**Table 4:** Multivariable regression analyses of the unique influence of individual ACEs on the total number of reported substance use in the past year based on a continuous scale.

	Substance use		Self-control	
	*β* [95% CI]	η^2^	*β* [95% CI]	η^2^
Individual ACEs (Entered simultaneously)				
1. Separation/Divorce	ns	**0.27**	ns	**0.30**
2. Emotional abuse	ns		ns	
3. Emotional neglect	ns		**−0.13 (95% CI: −0.24, −0.01)**	
4. Physical abuse	ns		Ns	
6. Household mental illness	**0.13 (95% CI: 0.03, 0.23)**			
5. Household substance abuse	**0.12 (95% CI: 0.02, 0.22)**		ns	
8. Sexual assault	**0.11 (95% CI: 0.01, 0.22)**		**−0.14 (95% CI: −0.24, −0.03)**	
7. Mother treated violently	ns		ns	
9. Incarcerated household member	ns		ns	
10. Physical neglect	ns		ns	
Three categories of ACEs (Entered simultaneously)				
3. Household dysfunction	**0.13 (95% CI: 0.02, 0.24)**	**0.24**	ns	**0.26**
2. Abuse	**0.12 (95% CI: 0.01, 0.23)**		**−0.21 (95% CI: −0.31, −0.10)**	
1. Neglect	**ns**		**−0.11 (95% CI: −0.21, −0.02)**	

*β*, Standardised beta; CI, Confidence Intervals; η^2^, Eta Squared (a measure of effect size). All significant associations are in bold.

### The influence of ACEs on self-control in univariable and multivariable regression models

The total number of ACEs generally significantly predicted self-control as a continuous measure (*β* = −0.35, 95% Confidence Interval [CI]: [−0.31, −0.08]). Except for physical abuse, household substance abuse, divorce/separation, and incarceration of a family member, every individual ACE strongly predicted self-control, albeit, differently (Table 3). The regression model produced a significant fit (*R*^2^ = 12.25, *F*_(2, 356)_ = 24.04, *p* < 0.001). Only household dysfunction, out of the three ACE categories of abuse, neglect, and dysfunctional households, significantly predicted self-control, upholding the hypothesis that ACEs cumulatively predicted substance use (Table 3). Again, including sex as a variable had no beneficial effect on the regression models. Each regression coefficient is the ratio of the SD change in the predictor variable to the SD change in the outcome variable. For instance, the regression of substance usage on the overall number of ACEs indicates a change of 1 SD associated with a change of 0.35 SD in substance use.

When all the ACEs were simultaneously included in a single regression model to assess their individual effects on self-control, only family mental illness, household substance use, and sexual abuse remained significant (Table 4). Similarly, only household dysfunction persisted as a significant predictor of self-control for the three ACE categories (neglect, abuse, and abuse; Table 4).

### The mediating effect of self-control in the relationship between ACEs and substance use in multivariable regression models

There was a statistically significant direct relationship between the total number of ACEs (as a continuous measure) and substance use when assessing the mediation model. The total number of ACEs as well as substance use were both significantly associated with self-control. Self-control mediated the relationship between ACEs and substance use, with a statistically significant indirect path (*β* = 0.21 [95% CI 0.12 to 0.22]) explaining 51.2 percent of the effect of ACEs on substance use. After the inclusion of self-control in the model, the effects of ACEs on substance use were considerably attenuated (*β* = 0.20 [95% CI: 0.10 to 0.30]), indicating partial mediation. Adding self-control to the mediation model improved the proportion of explained variance from *R*^2^ = 0.06 (*F*_(1, 537)_ = 30.91, *p* < 0.001) for the model with only ACEs to *R*^2^ = 0.11 (*F*_(2, 536)_ = 28.42, *p* < 0.001) for the model with both ACEs and self-control as predictors. In the present study, VIF were all less than 3.0, demonstrating that multi-collinearity had little effect on the outcomes.

## Discussion

### Main findings and corroboration with previous studies

Data from adolescent students in secondary schools in Uganda were analysed to determine the degree to which ACEs were linked to substance use. In agreement with the results of previous studies, more than 81 percent of adolescents reported experiencing one or more ACEs, and about 22 percent of them indicated that they experienced four or more ACEs (Amone-P’Olak, 2022). Further, the results of the current study generally concur with earlier research results on the prevalence and gender distribution of substance use (Dube et al., 2003; Fagan et al., 2015; Abbo et al., 2016; Rich et al., 2016; Olawole-Isaac et al., 2018; Amone-P’Olak and Letswai, 2020; Olashore et al., 2022). The most frequently reported types of ACEs were: physical abuse, emotional abuse and neglect, separation and divorce, and household substance abuse, in that order. Female students reported emotional and sexual abuse at rates that were significantly higher than those of male students, while male students reported significantly more physical abuse than their female peers (Amone-P’Olak and Letswai, 2020; Amone-P’Olak, 2022).

Furthermore, alcohol, codeine (cough syrup), cigarettes, khat, and marijuana were the most frequently used substances, in that order, according to the results of the current study. The number of female youths using substances is increasing, with potential sex differences being attributed more to chance rather than susceptibility (Etten et al., 1999; Etten and Anthony, 2001). For instance, a prior study of young adults also reported no significant difference in alcohol use between male and female students (Ludick and Amone-P’Olak, 2016). In the current study, there were no significant variations in alcohol, cocaine, and inhalant use by gender. However, male students reported significantly higher levels of tobacco, Khat, and opium use than their male peers female students used codeine (cough syrup) and Cozepam (Benzo Diazepam or Valium) significantly more than their male colleagues.

On the relationship between ACEs and substance use, only a few ACEs significantly predicted substance use in univariable analyses, suggesting that it may be exposure to multiple ACEs that are especially linked to substance use in young people (Shin et al., 2018). According to the results of the mediation model, self-control accounted for 51 percent of the associations between ACEs and substance use in support of the hypothesis that exposure to various childhood adversities may be linked to substance use in adolescence and early adulthood. The partial mediation implies that there may be additional contributing factors that lead adolescents and young adults to engage in substance such as peer pressure, the lack of adult supervision, poor stress management, and a poor school climate, among others. These factors will be addressed in future as they were outside the purview of the current study.

Previous studies showed that ACEs were associated with deficits in self-control (Shin et al., 2018; Meldrum et al., 2020). Consequently, both ACEs and self-control significantly predicted the use of substances (Tables 3, 4). After the addition of self-control in the mediation model, the relationships between prior ACEs and current substance use attenuated but remained significant. The current study adds to the literature on the effects of childhood maltreatment a growing corpus of research on correlates of substance use to include self-control and further supports the hypothesis that self-control mediates the associations between ACEs and substance use in adolescence and early adulthood.

**Figure 2:**
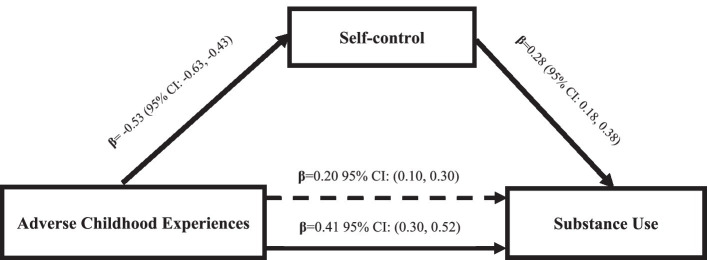
Mediation by Self-control of the relations between adverse childhood experiences and substance use. The coefficient immediately above the continuous line is before the mediator was added to the model and the one above the dotted line is after the mediator was added to the model. Indirect effect: (mediated effect) = *β* = 0.21, 95% CI: (0.11, 0.31). The proportion of total effect = 0.21/0.41 = 0.51.2 (or 51.2%). The ratio of indirect to direct effect = 0.21/0.20 = 1.05. The ratio of total to direct effect = 0.41/0.20 = 2.05. Bootstrap results = *β* = 0.63, 95% CI: (0.50, 0.76).

According to the results of the mediation model, self-control accounted for 51 percent of the associations between ACEs and substance use (Figure 2). The effects of ACEs on substance use attenuated but remained statistically significant, implying that there may be additional contributing factors that lead adolescents and young adults to engage in substance use. Substance use among adolescents and young adults may be influenced by a variety of other factors, including peer pressure, the lack of adult supervision, poor stress management, and a poor school climate, among others. These factors will be addressed in future as they were outside the purview of the current study.

### Meaning and implications of the results

In adolescents and young adults, there are two major possible pathways to substance use: psychosocial and neurobiological. Regarding psychosocial, indicators of ACEs, which include familial mental illness, substance abuse, and separation/divorce, can impair parental child-rearing practices, thus impairing offspring’s self-regulation and making it difficult for them to foresee the adverse long-term consequences of their behaviours. Consequently, the offspring will likely be prone to impulsiveness, self-centeredness, risk-seeking, and instant gratification, all typically deficient dysregulation that may easily predispose them to substance use (Gottfredson and Hirschi, 1990). Moreover, a family climate fraught with emotional neglect and abuse, physical abuse, and household substance abuse is often associated with poor rearing practices, poor parental control and monitoring, and a climate where rules and regulations are inconsistently enforced (Amone-P’Olak et al., 2009; Mongale and Amone-P’Olak, 2019; Phillip and Amone-P’Olak, 2019). For example, about 25 percent of the adolescents and young adults in this study reported parental separation or divorce. Separation and divorce are linked to poverty, stress, conflicts, and dysfunction, which ultimately results in deprivation, affects child-rearing practices, and makes young people vulnerable to future stress (Karatekin, 2018). Moreover, abuse, neglect, violence, substance abuse, and familial mental illness undermine social bonds in households fraught with ACEs. Young people raised in such households may lack self-control and may turn to substance use to relieve stress (Moitlakgola and Amone-P’Olak, 2015; Kgatitswe and Amone-P’Olak, 2017). This agrees with the SBT theory, which hypothesises that a breakdown of social bonds may predispose children to deviant behaviours (Hirschi, 1969a,b).

The neurobiological pathway may be another pathway from ACEs to substance use (Beers and De Bellis, 2002; Anda et al., 2006). According to the neurobiological theory, adverse childhood experiences result in dysfunctional brain development and suboptimal stress regulation (Beers and De Bellis, 2002; Anda et al., 2006). Stress is also known to hamper neurogenesis, which hinders brain maturation and development and may be linked to the dysregulation of the Hypothalamic–Pituitary–Adrenocortical (HPA) system (Beers and De Bellis, 2002; De Bellis et al., 2002; Anda et al., 2006; Thorpe et al., 2020) leading to adverse reactions to stress (Breedlove and Watson, 2013), which, in turn, make adolescents and young adults prone to substance use to cope with the stress (Kgatitswe and Amone-P’Olak, 2017).

### Limitations and strengths

The results of this study should be viewed in light of several limitations. First, retrospective reports are prone to recall bias (Fisher et al., 2011; Amone-P’Olak and Letswai, 2020). Nonetheless, only the number and not the timing of the ACEs or substance use were taken into account to reduce the likelihood of recall bias. The memory of when such events occurred is more prone to bias than the memory of whether they occurred at all. Second, because ACEs and substance use are stigmatised, it is probable that respondents found it difficult to report them leading to underreporting. Third, it is difficult to extrapolate the results to the larger non-school-going adolescents and young adults since the respondents were a homogenous group of school-going adolescents and young adults. Fourth, a comprehensive list of early childhood adversities such as HIV/AIDS disease, experiencing wars, bullying, community violence, poverty, or being orphaned, may not have been covered by the ACE-10 employed in this study. Furthermore, the DAST-10 list of substances was far from comprehensive, too. Last, but not least, it was not possible to establish cause and effect in this study due to the cross-sectional survey design. Likewise, the data source for ACEs, self-control and substance use were the same leading to the limitations of same-source variance. Same-source variance can lead to an underestimation of correlations in reliability estimates, which, in turn, may be artificially inflated (Fuller et al., 2016). Nevertheless, the current study checked for VIF, which was within normal ranges.

The strengths of the study may include the following: first, not many studies have been conducted on drug and substance use among adolescents in sub-Saharan Africa. Yet, the experience of childhood maltreatment and cumulative trauma is widespread in sub-Saharan Africa and exposes adolescents to the risk of drug and substance abuse. Second, the results of the current study add to the evidence base for practice and interventions in the sub-population of adolescents and young adults. Finally, we were able to study self-control as one of the factors that explain the relationship between ACEs and substance abuse and offer opportunities for intervention.

### Implications

Notwithstanding the limitations of the current study, the results may have several research, policy, and practice implications. First, there is an urgent need for research with longitudinal designs, and large samples from diverse subpopulations of adolescents and young adults to delineate the long-term consequences of childhood maltreatment and identify young people at risk of substance abuse (Amone-P’Olak and Letswai, 2020). Such studies would not only inform practice but provide the much-needed evidence to support interventions to reduce substance use in the subpopulations of adolescents and young adults. Second, although it was not a variable of the study, creating a supportive school environment that encourages healthy and adaptive coping with childhood adversity, reduces student stress, and offers guidance and counselling to decrease substance use is recommended. Finally, childhood maltreatment is a major concern that requires public health intervention. At the public health level, more health workers, psychologists, and social workers could be employed to continuously assess and monitor the well-being of children. For instance, as part of a child protection programme in primary health care, public health professionals could conduct assessments of every child in school once or twice a year until they are 16 years old (Amone-P’Olak and Letswai, 2020). This would enable early detection and remedy of any possible childhood maltreatment.

According to the Social Bonds Theory (SBT), ACEs reduce young people’s ability to self-regulate by weakening the SBT’s core tenets of attachment, commitment, engagement, and belief. Adolescents and young adults with a history of ACEs are thus more vulnerable to substance use as their social bonds are weakened by a plethora of ACEs. Subsequently, adolescents and young adults who are unable to maintain stronger social connections become impulsive, self-centred, risk-taking, and develop a desire for constant gratification, all of which erode their self-control rendering them susceptible to substance use.

## Conclusion

This study’s results indicate a link between ACEs and substance use. This link is partially mediated by self-control. Therefore, the risk for substance use in adolescents and young adults is partly explained by self-control. Overall, the results indicate that interventions that promote self-control may help to contribute to a reduction in substance use in adolescents and young adults exposed to ACEs.

## Data availability statement

The raw data supporting the conclusions of this article will be made available by the authors, without undue reservation.

## Ethics statement

The studies involving humans were approved by the Ethics Committee of Lira University Institutional Review Board. The studies were conducted in accordance with the local legislation and institutional requirements. Written informed consent for participation in this study was provided by the participants’ legal guardians/next of kin.

## Author contributions

JN: Conceptualization, Data curation, Formal analysis, Writing – review & editing. KA-P’O: Conceptualization, Data curation, Formal analysis, Writing – review & editing, Methodology, Writing – original draft. CN: Conceptualization, Formal analysis, Writing – review & editing. HK: Writing – review & editing. NM: Writing – review & editing. JS: Conceptualization, Formal analysis, Methodology, Writing – review & editing. BO: Conceptualization, Formal analysis, Methodology, Writing – review & editing.
